# Tuberculosis and Milk-Supply1Read before the American Public Health Association at the twenty-fifth annual meeting, Philadelphia, October 26-29, 1897.

**Published:** 1897-12

**Authors:** Mazyck P. Ravenel

**Affiliations:** Bacteriologist of the State Live-Stock Sanitary Board of Pennsylvania; Instructor of Bacteriology, Veterinary Department, University of Pennsylvania


### TUBERCULOSIS AND MILK-SUPPLY.[1]

By Mazyck P. Ravenel, M.D.,
BACTERIOLOGIST OF THE STATE LIVE-STOCK SANITARY BOARD OF PENNSYLVANIA; INSTRUCTOR OF BACTERIOLOGY, VETERINARY DEPARTMENT, UNIVERSITY OF PENNSYLVANIA.

As compared with other diseases to which man is liable, the one which must be recognized as deserving the greatest attention from sanitarians, health-officers, and physicians is unquestionably tuberculosis, and no apology is needed for the giving of any facts, however small, which may add to our understanding of it or our power of preventing it. Tuberculosis, according to the best statistics, causes one-seventh of all deaths in man. It has destroyed more lives than all the wars and epidemics of cholera, smallpox, and yellow fever combined. That it is on the decrease seems to be unquestionable, but it is a matter of the greatest importance to examine into the causes of its great prevalence.

Of all diseases to which the animals from which we derive our food are liable, tuberculosis is also the most prevalent and the most far-reaching in its effects, and it seems not unlikely that there is a close connection between the prevalence of the disease in man and in our food-animals. If we examine into the geographical distribution of tuberculosis we will find that there is a close connection between the presence and absence of tuberculosis and the presence and the absence of healthy cattle. In Northern Norway, Sweden, Lapland, and Finland, where reindeer constitute the bulk of farm-animals; or about the Hudson Bay and in the islands of the Pacific, where no cattle exist; in the Scottish Hebrides, Iceland, and Newfoundland, where there are only a few cattle, tuberculosis is far less prevalent in man. In Algiers the cattle are few, and live for the most part in the open air, away from cities, and it is found that tuberculosis does not increase among the natives. In Italy, on the other hand, where cattle are housed, Perroncito states that tuberculosis has become the scourge of man and beast (Law.)

No disease known attacks more numerous genera of animals than does tuberculosis. The bovine species are the most susceptible, but the disease is found in chickens, guinea-pigs, swine, rabbits and goats, and less frequently in caged apes, lions, kangaroo, deer, elk, gazelle, antelope, birds, and, in one case noted by Dr.

[1] Read before the American Public Health Association at the twenty-fifth annual meeting, Philadelphia, October 26–29, 1897.

Theobald Smith, a tame bear. Among the animals usually thought to be exempt, such as dogs, cats, sheep, and horses, the disease can readily be produced by inoculation.

It is, however, to the disease as found in milch-cattle that we wish to devote our especial attention in this paper. Professor Law states that in some dairy- and breeding-herds in New York, consisting largely of mature cows, there is found as high as 98 per cent. of tuberculous animals. In the State of Pennsylvania Professor Pearson has found herds in which every animal was afflicted by the disease. The disease, probably, did not prevail among American cattle to any extent, at least, until comparatively recent years. It has probably been developed by in-and-in-breeding, over-milking, breeding too young, overtaxing, and other weakening effects, as the effort to supply the increasing demand for milk in our rapidly growing American cities has become greater; although, no doubt, the chief means of increase has been by direct infection from animal to animal.

The statistics of 1890 show that the number of cows in the United States was 15,952,883, yielding a total of 6,750,000,000 gallons of milk, or about 100 gallons per capita for each inhabitant. Of this amount of milk only 5 per cent. is used as butter and cheese, the other 95 per cent. being consumed as milk in its natural state. If milk can act as a carrier of the tubercle bacillus what a fruitful source of the disease this enormous supply must be! It has also been found in butter by numerous observers, and has been shown to maintain its life in that substance for upward of one hundred and twenty days; likewise, cheese has been found to contain it, and it is known to live in this substance for as much as thirty-five days. So even the manufactured products of milk may convey the disease, and we can look upon the total amount of milk produced as a possible conveyer of the tubercle bacillus.

The danger from milk was first pointed out by Professor Klencke as early as 1846, who gave the clinical histories of sixteen children who had been fed from the milk of cows, some of which were stable-fed and some swill-fed, all of which pointed to tuberculosis either of the intestines, glands, skin, or bone. This virulence of milk was confirmed by Gerlach in 1869, and later by others. The discovery of the tubercle bacillus by Koch in 1882 proved the possibility of what these men had found to be true clinically, and in the same year both Virchow and himself pointed out the possibility of the danger of infection of milk by the tubercle bacillus.

Among those who have given further clinical evidence of infection by means of milk may be mentioned Dr. Stang, of Amorback, who mentions a case of a well-developed five-year-old boy from sound parents, whose ancestors on both sides were free from hereditary taint, who died after a few weeks' illness of acute miliary tuberculosis of the lungs and enormously enlarged mesenteric glands. A short time before the parents had had a milch-cow killed and found her the victim of advanced tuberculosis. Brouardel cites a case where five out of fourteen young girls living together in a boarding-school became consumptive subsequent to the daily use of milk from a tuberculous cow (Pearmain and Moor.)

Dr. Demme records a case of four infants in the Child's Hospital at Berne, without tuberculous ancestors, who died of intestinal and mesenteric tuberculosis, as the result of feeding on the unsterilized milk of tuberculous cows.

Professor Law states that after a lecture at Providence, Rhode Island, a gentleman of North Hadley, Mass., publicly stated that his only child, a strong, vigorous boy of one and a half years, went to an uncle's for one week and drank the milk of a cow which was shortly afterward condemned and killed in a state of generalized tuberculosis. In six weeks the child was noticeably falling off, and in three months died of tuberculosis of the abdomen. There was no tuberculosis on the father's side, but some on his mother's, although she herself was in perfect health.

Dr. E. O. Shakespeare attributes one-fifth of all deaths in infants and young children feeding on milk to tuberculosis, usually commencing in some part of the digestive organs.

Woodhead has recently stated "that from his experience in two large hospitals he has been much struck by the fact that in children who had died from other diseases during the course of tubercular disease of the abdominal gland there was frequently not any trace of tubercular disease in any part, thus pointing to the intestine as a channel by which the bacillus made its way into the body." He also remarks that in a large number of cases of general tuberculosis, where the possibility of infection by the pulmonary passages was evidently excluded, the tuberculous process appeared to have invaded the body by the intestinal canal. These facts, taken in connection with the occasional existence of tubercle bacilli in milk, went far to prove in his opinion that milk was a source of tubercular infection, especially in young children. Woodhead found that out of 127 cases of tuberculosis in children the mesenteric glands showed tubercular infection in 100, and that there was ulceration of the intestine in 43. It is especially in children that this mode of infection occurs. In the adult, ulceration of the intestine is rare as a primary infection.

Direct evidence of the infection of adults by means of milk is wanting, and we should expect to find in such cases that primary tuberculosis of the intestine would be a common manifestation of the disease; but such is not the fact as shown by clinical evidence. That such infection does not take place in adults more frequently may, perhaps, be accounted for to some extent, at least, by the fact that a strong, vigorous digestion seems in some measure to protect the consumer. Peuch fed a two-months pig in five days four and a half quarts of milk drawn from a tuberculous udder, and when killed after fifty-six days it was found quite sound. Four rabbits inoculated with the same milk all became tuberculous.

The infection of animals by means of tuberculous milk, either by ingestion or by inoculation, is beyond question, different observers all agreeing on the main fact, their percentages of successful inoculation merely being different. It was formerly taught, and is still held by some experimenters, that the milk does not contain the tubercle bacillus, and is therefore not infectious, unless there is coincident disease of the udder of the cow producing it. There is, however, very strong evidence against this opinion. Ernst found in 33 per cent. of the cows examined by him, in which a careful post-mortem examination was made, which showed that the animals were widely affected with tuberculosis, but that the udder was free from disease, that the milk contained the tubercle bacillus. In 1893 Theobald Smith showed that the tubercle bacillus may be present in the milk of tuberculous cows when the udder, so far as the naked eye could tell, was free from disease. Other observers have given similar results.

The most positive evidence, however, is given by inoculation-experiments. Hirschberger, by inoculation of rabbits in the abdominal cavity with the milk of 29 tuberculous cows, of which the udders were, or appeared, sound, produced tuberculosis 14 times. Bang inoculated from 63 tuberculous cows selected for their sound udders, and found the milk of 9 of them infectious. A subsequent microscopic examination showed the udders of 3 of these cows to be diseased, leaving 6 which gave infectious milk in which even after death no tubercle bacilli could be found in the udder. Ernst found 10 cows in 35 with infecting milk, though the udders were sound. Of 103 animals inoculated, 17 contracted tuberculosis, and of 12 calves sucking the cows, 5 became tuberculous. Drs.

Smith and Kilborne found the milk infecting in 3 cows out of 6 with apparently healthy udders. Forty-four per cent. of the guinea-pigs inoculated contracted tuberculosis: 1 in 5 from one cow, 8 in 10 from another, and 6 in 6 from a third. Professor Law gives his experience that 3 calves from healthy parents, sucking the apparently sound udders of 3 cows with general tuberculosis, all contracted the disease. Galtier found, by inoculation, that milk may prove infectious in 60 per cent. of cases, and that the infectious qualities are greatest in lesions of the udder, and next with those affected with general tuberculosis. Baumgarten, Fisher, and Wesener, in their food-experiments were especially impressed with the resulting tubercular lesions of the intestinal mucosa, mesenteric glands, and liver.

It is also known that the primary seat of tuberculosis in children is so frequently in the intestines and their related glands, and milk forms so large and essential an element in the food of children, that we cannot avoid the conclusion that it is to them a frequent source of infection.

Dr. Russell, Health-officer of Glasgow, is so impressed with the danger of tubercular infection by milk that he regards the supervision of the milk-supply as of at least equivalent importance with the regulation of the expectoration of consumptives and as being a much more practicable matter of sanitary administration. He says: "There is no need for argument in reference to the milk of tuberculous cows. The facts are so universally accepted, and so grave, that the administrative effects shine through them. It is practically the Glasgow position—that a tuberculous cow must not be retained in a dairy. There is a remarkable consensus of opinion as to the influence of milk in disseminating tuberculosis, especially among the young. The pathologist finds that of the total deaths under ten years of age among the mass of people, about a third are due to tuberculosis, and the usual seat of the disease at that age points to food as the medium of infection. The prevalence of tuberculosis among dairy cows is notorious."

Dr. Martin, in the report of the Royal Commission on Tuberculosis, writes: "The milk of cows with tuberculosis of the udder possesses a virulence which can only be described as extraordinary; all the animals inoculated showed tuberculosis in its most rapid form." Dr. Woodhead, investigating for his own purposes the effects of unboiled milk, speaks in similar terms of this virulence of milk derived from tuberculous udders and inoculated into test-animals.

These two observers examined the milk from a cow in which one-quarter only of the udder showed tubercular disease. They found the milk from the other three-quarters perfectly harmless on inoculation, but the milk taken from the four teats was to all appearances just as virulent as the milk from the diseased quarter. Butter, skim-milk, and buttermilk obtained from the milk of a cow with tuberculous udder all contained tuberculous matter, actively injurious to test-animals. Dr. Woodhead calls attention in the same report to the rapidity with which tuberculosis of the udder spreads, and states that in cows constantly under observation he had noticed on several occasions during the interval between the fortnightly inspections carried on along with a veterinary surgeon, that the disease had become distinctly developed. He says: "It may be, of course, that the early evidence has been overlooked at the previous inspection; but whether this is the case or not the spread of the disease is so rapid as to afford very good ground for alarm. The very absence of any definite sign in the earlier stage is one of the greatest dangers of this condition." Both Dr. Martin and Dr. Woodhead insist that no tuberculous animal of any kind be allowed to remain in a dairy.

The identity of tuberculosis in cattle and in man seems to be beyond question, although the manifestations of it may differ at times to a great extent. A few cases showing dircet transferrence of the disease from cattle to man by inoculation may be quoted.

Tscherming, of Copenhagen, attended a veterinarian who had cut his finger in making a post-mortem examination upon a tuberculous cow. After healing, the wound began to ulcerate, and the finger was finally removed. Microscopic examination revealed a tuberculous process with the presence of the tubercle bacilli.

Pfeiffer attended a veterinarian thirty-four years of age, with a good constitution and without hereditary predisposition, who cut his thumb in making a post-mortem examination of a tuberculous cow. The wound healed, but six months later the cicatrix still remained swollen, and in the fall of the next year the man developed pulmonary tuberculosis, bacilli were found in his sputum, and death occurred two and a half years after the wound. Post-mortem examination revealed tuberculosis of the joint of the wounded thumb.

Professor Law quotes a case of a young veterinarian, a friend of his, who was inoculated in the hand while opening a tuberculous cow, and who suffered from a tumefaction of the resulting cicatrix, which showed tubercle bacilli.

To these cases may be added one which came under the observation of the writer recently. Dr. E., a veterinarian of Downingtown, Pa., cut the knuckle of his forefinger while making a post-mortem examination of a tuberculous cow. The wound healed badly, remained swollen, and showed decided tendency to ulcerate. Removal of the cicatricial mass was practised and the tissues sent to me for examination. They showed typical tubercular lesions, with giant-cell formation.

Further evidence is given by the condition of our Northwest Indians. Dr. Holder, in the *Medical Record*, gives the Indian mortality from consumption as 50 per cent. of all deaths, at Green Bay, Wis., Tulalip, Wash., and Western Shoshone, Nev. He says that at Lower Brulé, Dak., scrofula is present in 60 per cent. of the Sioux under twenty-one years, and that at Crow Creek, Dak., 50 out of a total Indian population of 1200 die yearly of consumption and scrofula. The animals furnished to these tribes are said by Dr. Treon, an American practitioner, to be poor, emaciated, and diseased. He describes how they eat liver, tallow, and entrails, raw and fresh, and how the carcass is dried, pounded, and packed in the skins to be eaten later without cooking. The meat is eaten, even though the animal may have died of disease. (Law.)

Although it is manifestly impossible to try direct inoculation from animal to man, corroborative evidence may be had by inoculating animals from man. This has been done in numerous cases, only one of which, given by Professor Crookshank, will be quoted. He obtained sputum containing numerous bacilli from an advanced case of phthisis, the sputum was shaken up with sterilized salt-solution, and injected into the peritoneal cavity. The calf showed illness in a few weeks, did not feed well, and had a slight cough. These symptoms increased, and death occurred forty-two days after inoculation. Extensive lesions were discovered at the post-mortem examination ; extending over the mesentery from about the point of inoculation were hundreds of wart-like, fleshy new-growths. There were similar deposits on the under surface of the liver, the spleen, in the gastro-splenic omentum, and on the peritoneal surface of the diaphragm. Microscopical examinations of the sections revealed minute tubercles disseminated through the whole section of the lungs and liver, and tubercle bacilli were found in these and in the peritoneal deposits. The calf died of pyæmia, but sufficient time had elapsed for marked local infection leading to generalized miliary tuberculosis.

For the past year the writer has carried out experiments bearing on the subject of milk-infection, under the auspices of the Live-stock Sanitary Board of the State of Pennsylvania at the Veterinary Department of the University of Pennsylvania. Five grade-cows which reacted to tuberculin and showed physical signs of tuberculosis were obtained, the udders in every case being free from disease as far as a careful inspection could reveal. The cows during the experiments were kept under the best sanitary condition, were carefully fed, groomed every day, and given as much outdoor life in a paddock in the yard as possible. Guinea-pigs were used exclusively, and the method employed was intraperitoneal injection. The milk was in no case centrifugalized; on the contrary, the whole milking was thoroughly shaken together before being used, the idea being to approach as nearly as possible the conditions which would be obtained from ordinary dairy milk. A single dose averaging 10 cubic centimetres was given.[1] The results were as follows:

SERIES A. Fifty pigs were used in this experiment, eighteen of which died within a few days of the injection, too soon for any signs of tuberculosis to have manifested themselves, and one was lost. Subtracting these, and considering only those which lived long enough for the disease to have become manifest, we have left thirty-one animals, four of which were unquestionably tuberculous, a percentage of 12.9.

SERIES B. Thirty pigs were used in this experiment, six of which died before tubercular lesions could have become manifest. Subtracting these, we have left twenty-four animals, five of which were unquestionably tuberculous, a percentage of 20.8.

SERIES C. Eight pigs were used in this experiment, and the milk of a single cow was employed. One pig became tuberculous, a percentage of 12.5. Eighty-eight animals were employed in these three series of experiments. Deducting those which died too soon after inoculation to show tubercular lesions, we have as a final result that 15.4 per cent. of the animals became tuberculous from the single dose of milk. In all cases the most healthy pigs were selected, and the experiment ended with the death and careful post-mortem examination of the animal.

SERIES D. In this experiment fifty-two pigs were used. Two were inoculated daily with mixed herd-milk; and two with the same milk after sterilization by heat. None of these animals became tuberculous. It has not seemed proper to include this

[1] The tables will be published in full in the Journal of the American Public Health Association. Only the final results are here given.

series with the others, but the figures are given for the sake of fairness, and also because of the interest they bear. When this experiment began the cows had been under treatment about eight months, the treatment consisting in the use of large injections of tuberculin (5 to 10 c.c.) on an average of every three weeks. All had improved very much, and one had gained more than 100 pounds in weight. In these experiments it must be remembered that no attempt was made to collect the bacilli in a small quantity of fluid, but, on the other hand, they were distributed as thoroughly as possible before the inoculation.

From these and other like experiments it is fair to conclude that the number of bacilli in the milk of tuberculous animals varies from day to day, although it is possible that in taking 10 c.c. from the whole mass of milk, we may have missed bacilli which were few in numbers. Additional evidence has also been given to show that the bacillus of tuberculosis may pass into the milk of cows having general tuberculosis, but whose udders are perfectly healthy, so far as the most careful examination by competent veterinarians can show.

I have given in these pages only a part of the evidence that is at hand on the question; but enough has been said, I think, to show the soundness of the conclusions arrived at by the Royal Commission on Tuberculosis, that,

. . . . "As to the proportion of tuberculosis acquired by man through his food or through other means, we can form no definite opinion, but we think it probable that an appreciable part of the tuberculosis that affects man is obtained through his food."

And "No doubt the largest part of the tuberculosis which man obtains through his food is by means of milk containing tuberculous matter."

The remedy lies in the careful inspection of milch-cows and the immediate weeding out of all diseased animals found. Milk from suspected cattle should be carefully sterilized before using, and especially should not be given to infants and invalids. The inspections of the animals should be at intervals frequent enough to keep the disease from gaining headway before being discovered.

---

McKillip Veterinary College reports a total number of students of thirty-two in the several classes. This is a very gratifying increase over the past two years, and points strongly to the increased interest in veterinary matters in the West.

